# Heavy metal burden in the urine and cancer risk

**DOI:** 10.3389/fonc.2025.1545118

**Published:** 2025-07-23

**Authors:** Hongqian Ke, Ning Xie, Qian Wu, Yuling Ye, Yi Fang

**Affiliations:** ^1^ Department of Gynecology, Clinical Oncology School of Fujian Medical University, Fujian Cancer Hospital, Fuzhou, Fujian, China; ^2^ College of Clinical Medicine for Oncology, Fujian Medical University, Fuzhou, Fujian, China; ^3^ Department of Radiation Oncology, Fudan University Shanghai Cancer Center Xiamen Hospital, Xiamen, China

**Keywords:** cadmium, cobalt, lead, cancer, NHANES

## Abstract

**Aims:**

The purpose of this study was to evaluate the association between exposure to urinary heavy metals and cancer risk in adults in the United States.

**Methods:**

The statistical data for this study were obtained from the National Health and Nutrition Examination Survey (NHANES) spanning 2009-2018. Participants lacking complete data on urinary heavy metals exposure burden and/or cancer status information were excluded. Urinary heavy metal concentrations were quantified using inductively coupled plasma mass spectrometry (ICP-MS). Cancer diagnoses were ascertained through self-reported medical histories. Multivariable-adjusted regression analyses and cubic smoothing plots were employed to assess independent associations between urinary heavy metal concentrations and cancer risk. Subgroup analyses were performed to assess result robustness.

**Results:**

The study included 7797 participants. Based on the first quartile (Q1), cadmium quartiles showed odds ratios (95% CIs) of 1.20 (0.92, 1.66) 1.50 (1.16, 1.94) and 1.57 (1.22, 2.03) for cancer prevalence (P = 0.0008). Cobalt quartiles were 1.22 (0.98, 1.54), 1.24 (0.98, 1.56), and 1.43 (1.13, 1.80) compared to the first quartile (Q1) (P = 0.0053). In comparison with the first quartile (Q1), Lead quartiles were 0.99 (0.77, 1.27), 1.06 (0.83, 1.35), and 1.06 (0.83, 1.35) (P = 0.0011). In the RCS plot, the association between log_2_-transformed urinary metal levels and cancer risk was not linear(P<0.05). An analysis of subgroups confirmed the robustness of the results.

**Conclusion:**

Elevated urinary heavy metal concentrations among U.S. adults demonstrated a significant association with increased cancer risk. These findings suggest that mitigating exposure to urinary heavy metals should be prioritized as a preventive strategy for cancer control.

## Introduction

According to the Global Cancer Observatory (GLOBOCAN) 2022 estimates, approximately 20 million new cancer cases and 9.7 million cancer-related deaths were documented globally in 2022 (1). It is estimated that one in five men and women will develop cancer at some point in their lives. These statistics underscore the critical need for implementing evidence-based preventive interventions to mitigate the global cancer burden.

Since heavy metals are non-biodegradable, they remain in the body for a longer period of time and pose a long-term health risk. The oxidative stress induced by toxic metals, cell growth stimulation, genomic instability, and alteration of cell proliferation have been well established (2–4).

Cadmium (Cd), cobalt (Co), and lead (Pb) are typical environmental pollutants, and numerous studies have confirmed their association with carcinogenicity. In Korean men, at the current level of exposure, blood lead concentrations were associated with prostate cancer risk (5). Soha et al. found that colon cancer patients had higher levels of Pb and Cu in their blood than healthy people, suggesting that high Pb levels may contribute to colon cancer (6). The serum levels of Cd and Mn were positively related to the risk of nasopharyngeal carcinoma in a study by Soha et al. (7).

In spite of this, previous studies still have some limitations. The majority of previous studies have focused on heavy metal exposure and its link to a single type of cancer (7–9). Then, heavy metal concentrations in the air or blood have been used in most studies to measure the risk of cancer associated with metal exposure (10, 11). Besides, it is unclear how heavy metal exposure affects cancer risk.

In the first part of our study, we analyzed the effect of multiple heavy metals on tumor incidence. Our study measured exposure in a different way by quantifying the concentration of metals in urine, which is the simplest and most non - invasive method available.

In the National Health and Nutrition Examination Survey (NHANES), quantitative methods are used to analyze urine specimens from subjects suspected of being exposed to a number of important metal elements. Accordingly, NHANES (2009–2018) data were used to investigate the association between metal concentrations in adults' urine and cancer.

## Materials and methods

### Population

**Figure 1:**
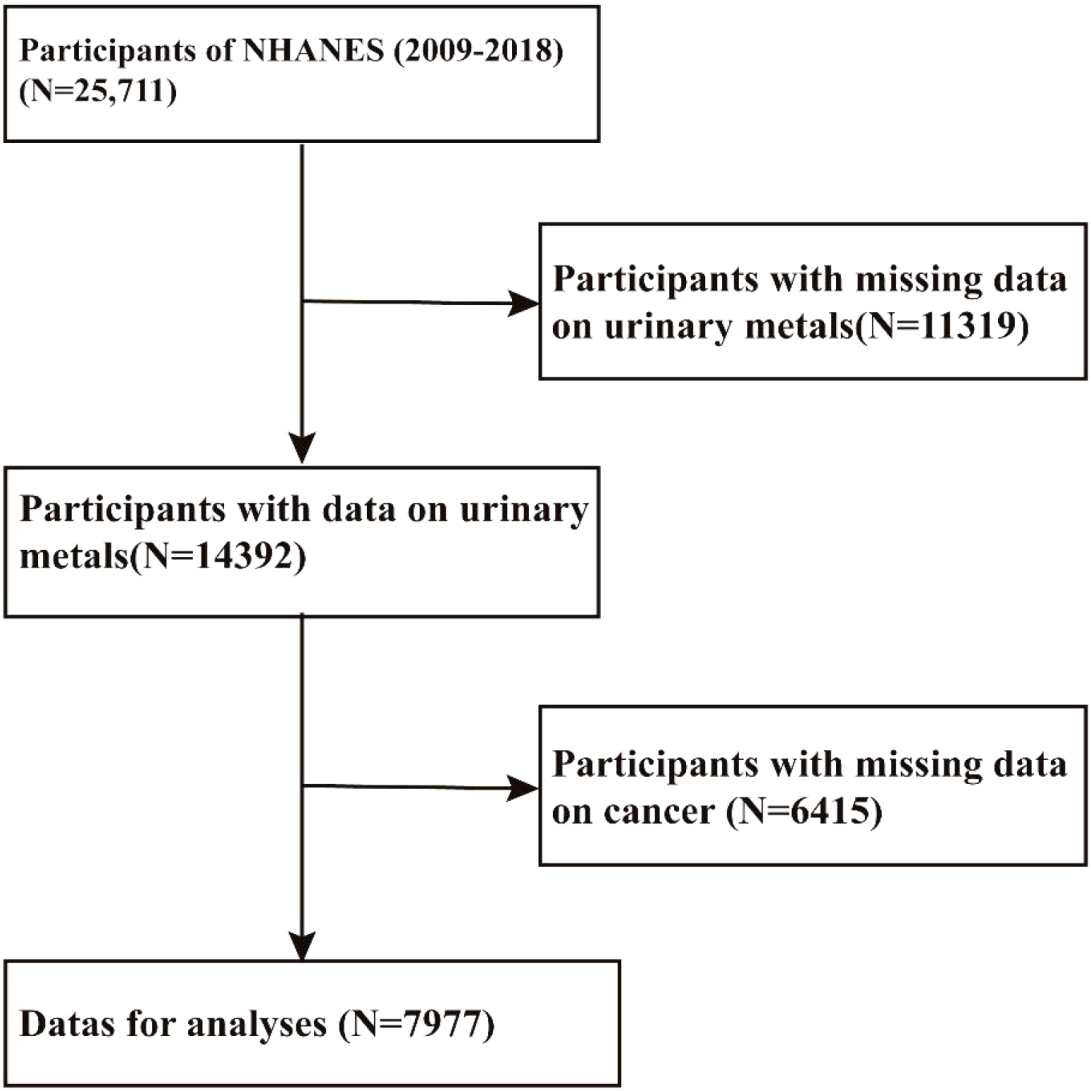
Flow chart of the study population. NHANES: the National Health and Nutrition Examination Survey.

In this study, NHANES data were used, an open national survey conducted by the National Center for Health Statistics. Stratified, multistage probability samples of individuals are selected from the general population through a complex statistical process to represent civilian non-institutionalized residents. An interview covers demographic, socioeconomic, dietary, and health-related topics. Its data will be used in epidemiological and health sciences research and can be made public on the NHANES website (https://www.cdc.gov/nchs/nhanes/index.htm). A detailed description of the study design, survey methods, population, and data is available on the website. NHANES data for ten years (2009 - 2010, 2011–2012, 2013–2014, 2015–2016, 2017 - 2018) were aggregated. Among the 25,711 adult participants, 17,734 had no information regarding urinary metal levels or cancer - related outcomes. Finally, 7,977 U.S. adults were enrolled for the analysis of the association between urinary metal concentrations and cancer (**Figure 1**).

### Exposure and outcome definitions

NHANES (2009–2018) urine samples contain metals such as Cadmium, Cobalt, and Lead. After confirming that the collection materials were free from contamination, a casual (or spot) urine specimen was collected from each participant. Inductively coupled plasma mass spectrometry (ICP-MS) was used to detect urinary metal levels. ICP-MS is a multi-element analytical technique capable of analyzing trace levels of elements. A nebulizer reduces liquid samples to small droplets in an argon aerosol and introduces the droplets into the mass spectrometer through the ICP ionization source. In order to determine individual isotopes of an element, the ions pass first through a focusing region, then through the DRC and then the quadrupole mass filter. Upon receipt, urine samples were stored at –30°C until analysis. A detailed procedure manual can be found online (CDC, 2018). A history of cancer was determined based on self-reported physician diagnosis (yes/no).

### Covariates

Medical professionals administered uniform interviews, physical examinations, and questionnaires to obtain covariates. Covariates in our study included gender (male/female), age (years), race (Mexican American/other Hispanic/non-Hispanic White/non-Hispanic Black/other races), education level (less than high school/high school or general educational development/above high school), body mass index (BMI), hypertension, diabetes status, and ratio of family income to poverty (PIR). BMI was calculated as weight divided by the square of height (kg/m²). Self-reported histories of hypertension and diabetes mellitus were used to determine the presence of hypertension and diabetes. The detailed measurement processes of the study variables are available at www.cdc.gov/nchs/nhanes/.

### Statistical analysis

Categorical variables were presented as percentages, while continuous variables were presented as means with standard errors (SE). An evaluation of the differences in groups divided by cancer was performed using either a weighted Student's t-test or a weighted Chi-square test. The relationship between urinary metals and cancer prevalence was analyzed using multivariate logistic regression models and 95% confidence intervals (CI). The lowest quartile (Q1) of each urinary metal served as the reference group. In Model 1, no covariates were adjusted. Model 2 was adjusted for age, gender, and race. Model 3 was adjusted for gender, age, race, education level, BMI, hypertension, PIR, and diabetes status. To test the heterogeneity of associations between subgroups, an interaction term was added. The heterogeneity of associations across subgroups was assessed using the Likelihood Ratio Test (LRT). The linearity/non-linearity of the relationship between log2-transformed urinary metals and cancer risk was tested using a smooth plot.

P <0.05 was considered statistically significant. Empower software (www.empowerstats.com;X&Y solutions, Inc., Boston MA) and R version 4.1.2 (http://www.R-project.org, The R Foundation) were used for all analyses.

## Results

### Participants

A total of 7,977 participants were enrolled in the study from the NHANES 2009 to 2018 cycles (**Figure 1**).

The characteristics of the study population are shown in **Table 1**. Compared with the non-cancer group, the participants with cancer were older and had a higher prevalence of diabetes and hypertension. The distribution of race and PIR differed between the cancer and non-cancer groups (p < 0.05). No significant difference in gender, education levels, and BMI was observed between the two groups (p > 0.05). The mean cadmium level was 0.35 ± 0.44 µg/l in the non-cancer group and 0.42 ± 0.44 µg/l in the cancer group, respectively. The mean cobalt level was 0.54 ± 1.08 µg/l in the non-cancer group and 0.64 ± 1.50 µg/l in the cancer group. The mean lead level was 0.58 ± 1.08 µg/l in the non-cancer group, while in the cancer group, it was 0.74 ± 1.19 µg/l.

**Table 1:** Participants’ baseline characteristics.

Variables	Non-cancer	Cancer	P
	(N=7234)	(N=743)	
Age(year)			<0.001
< 49	3825 (52.88%)	105 (14.13%)	
≧49	3409 (47.12%)	638 (85.87%)	
Gender			0.929
Male	3537 (48.89%)	362 (48.72%)	
Female	3697 (51.11%)	381 (51.28%)	
Race			<0.001
Mexican American	1094 (15.12%)	57 (7.67%)	
Other Hispanic	760 (10.51%)	43 (5.79%)	
Non-Hispanic White	2742 (37.90%)	498 (67.03%)	
Non-Hispanic Black	1553 (21.47%)	102 (13.73%)	
Other Race	1085 (15.00%)	43 (5.79%)	
Education			0.068
Less than high school	1644 (22.73%)	142 (19.11%)	
High school or GED	1641 (22.68%)	170 (22.88%)	
Above high school	3949 (54.59%)	431 (58.01%)	
BMI (kg/m2)	29.30 ± 7.09	29.08 ± 6.52	0.426
Hypertension			<0.001
Yes	2455 (33.94%)	429 (57.74%)	
No	4779 (66.06%)	314 (42.26%)	
Diabetes			<0.001
Yes	896 (12.39%)	152 (20.46%)	
No	6157 (85.11%)	555 (74.70%)	
Borderline	181 (2.50%)	36 (4.85%)	
PIR			<0.001
≦ 1.30	2591 (35.82%)	204 (27.46%)	
1.3-1.85	1139 (15.75%)	109 (14.67%)	
PIR			
> 1.85	3504 (48.44%)	430 (57.87%)	
Cadmium(µg/l)	0.35 ± 0.44	0.42 ± 0.44	<0.001
Cobalt(µg/l)	0.54 ± 1.08	0.64 ± 1.50	0.024
Lead(µg/l)	0.58 ± 1.08	0.74 ± 1.19	<0.001

A weighted mean (weighted SD) or weighted frequency (weighted percent) is presented for data. The data were gathered from the National Health and Nutrition Examination Survey 2009-2018.

### Associations between urinary metals and cancer

Under three models, multivariate logistic regression analysis was used to examine the associations between urinary metals, including cadmium, cobalt, and lead, and cancer risk. In Model 1, no parameters were adjusted. Model 2 adjusted for age, gender, and race. In addition to the variables adjusted for in Model 2, Model 3 further included education level, BMI, hypertension, diabetes status, and PIR (**Table 2**).

**Table 2:** Multivariate logistic regression models of the risk of cancer with urinary metals.

Subgroups	Model 1	Model 2	Model 3
		(OR, 95%CI)	
Cadmium			
Q1	Ref.	Ref.	Ref.
Q2	1.49 (1.16, 1.92)	1.19 (0.92, 1.55)	1.20 (0.92, 1.56)
Q3	2.06 (1.63, 2.62)	1.48 (1.15, 1.90)	1.50 (1.16, 1.94)
Q4	2.41 (1.91, 3.04)	1.51 (1.18, 1.94)	1.57 (1.22, 2.03)
P for trend	<0.0001	0.0026	0.0008
Cobalt			
Q1	Ref.	Ref.	Ref.
Q2	1.25 (1.01, 1.55)	1.23 (0.98, 1.54)	1.22 (0.98, 1.54)
Q3	1.13 (0.91, 1.41)	1.20 (0.95, 1.51)	1.24 (0.98, 1.56)
Q4	1.20 (0.96, 1.49)	1.40 (1.11, 1.76)	1.43 (1.13, 1.80)
P for trend	0.3025	0.0092	0.0053
Lead			
Q1	Ref.	Ref.	Ref.
Q2	1.15 (0.91, 1.46)	0.99 (0.78, 1.27)	0.99 (0.77, 1.27)
Q3	1.31 (1.04, 1.64)	1.01 (0.79, 1.28)	1.06 (0.83, 1.35)
Q4	1.79 (1.44, 2.22)	1.26 (1.00, 1.60)	1.06 (0.83, 1.35)
P for trend	<0.0001	0.0127	0.0011

Urinary metal levels were converted to a categorical variable (quartiles). OR stands for odds ratio, and 95% CI stands for 95% confidence interval. Parameters were not adjusted in Model 1. In Model 2, age, gender, and race were adjusted. In Model 3, gender, age, race, education level, BMI, hypertension, diabetes status, and PIR were adjusted.

In Model 3, the prevalence of cancer was significantly and positively related to urinary metals (**Table 2**). Based on the first quartile (Q1), the odds ratios (95% CIs) for cadmium quartiles were 1.20 (0.92, 1.66), 1.50 (1.16, 1.94), and 1.57 (1.22, 2.03) for cancer prevalence (P for trend = 0.0008). Compared with the first quartile (Q1), the odds ratios for cobalt quartiles were 1.22 (0.98, 1.54), 1.24 (0.98, 1.56), and 1.43 (1.13, 1.80) (P = 0.0053). Compared with the first quartile (Q1), the odds ratios for lead quartiles were 0.99 (0.77, 1.27), 1.06 (0.83, 1.35), and 1.06 (0.83, 1.35) (P = 0.0011).

### Subgroup analysis

**Figure 2:**
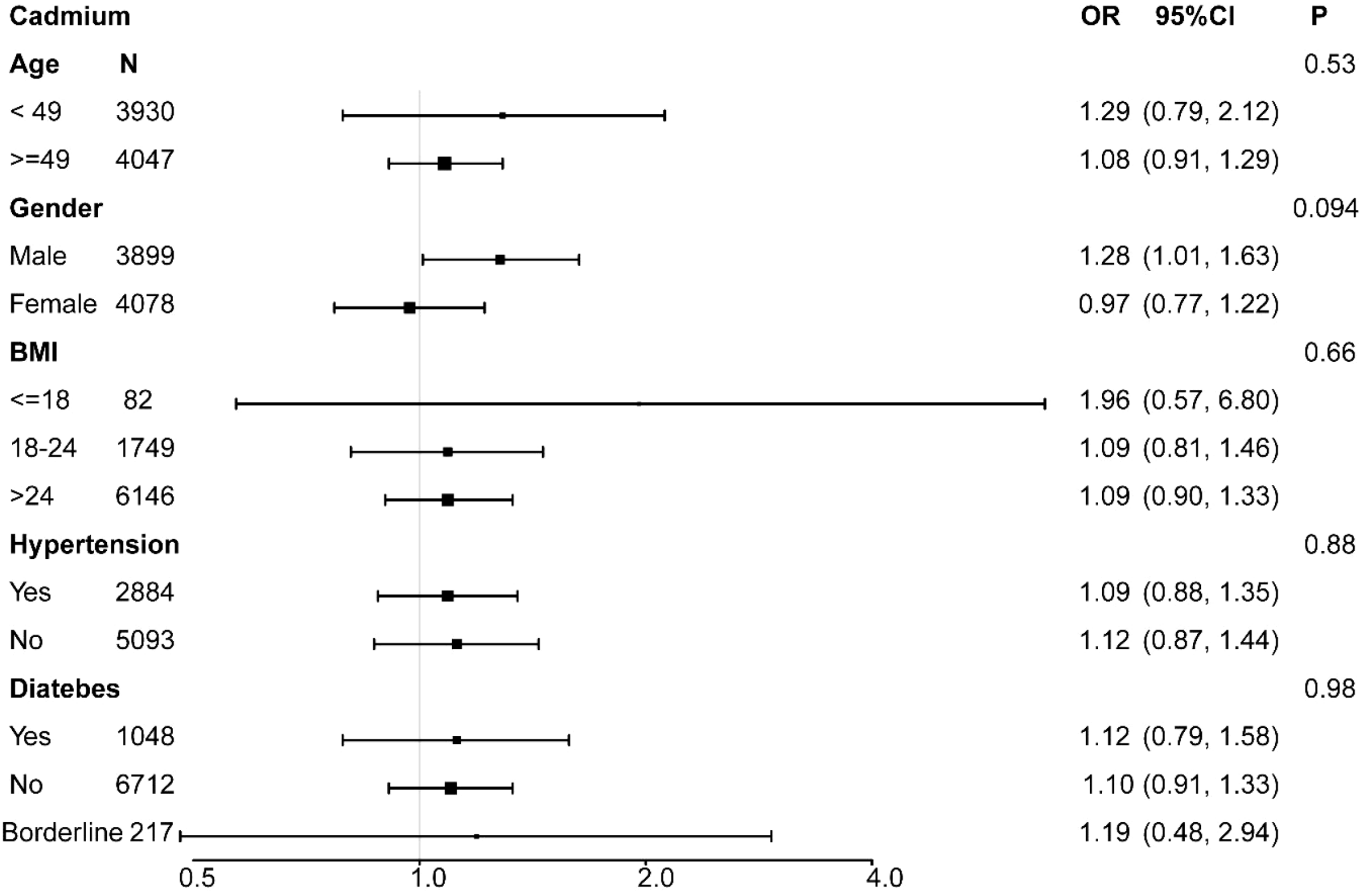
Subgroup analysis for the association between urinary cadmium concentration and cancer risk.

**Figure 3:**
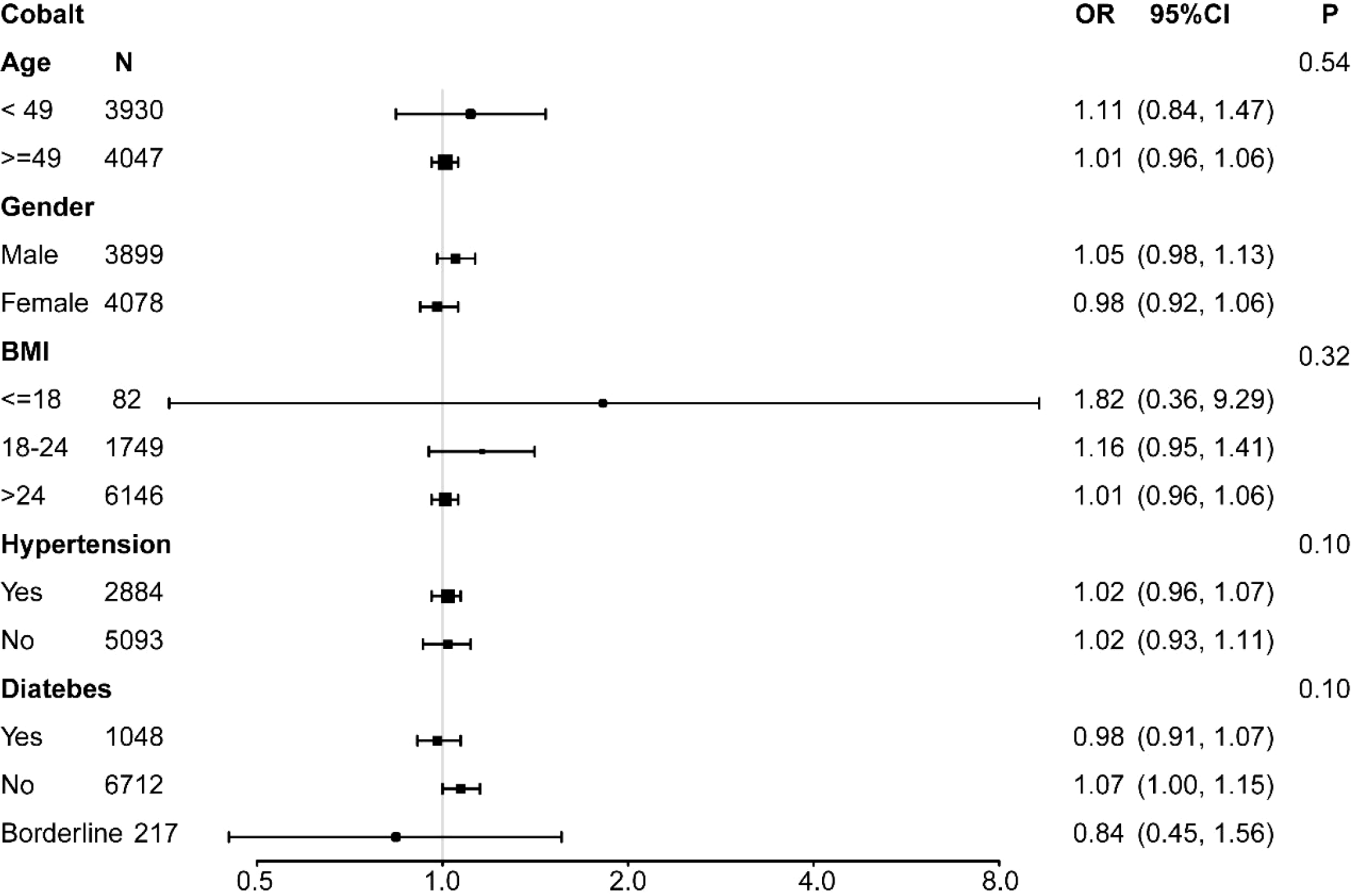
Subgroup analysis for the association between urinary cobalt concentration and cancer risk.

**Figure 4:**
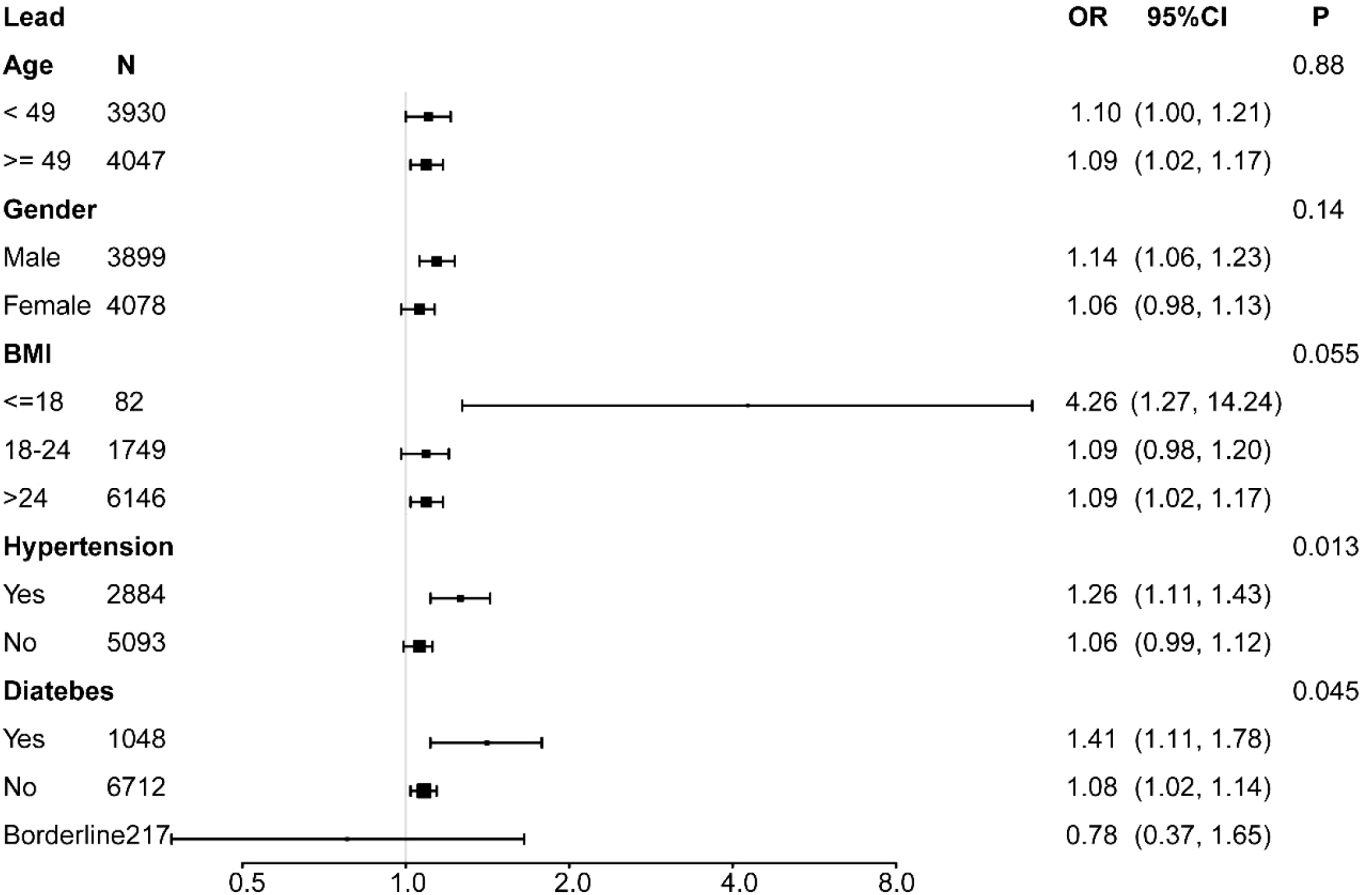
Subgroup analysis for the association between urinary lead concentration and cancer risk.

For further evaluation, a subgroup analysis was conducted. Age, gender, BMI, hypertension, and diabetes were included in an interaction test. In the cadmium group, the p-values for interaction did not reach statistical significance, indicating that the association was independent of age, gender, BMI, hypertension, and diabetes (all p > 0.05) (**Figure 2**). Similar results were observed in the cobalt group (all p > 0.05) (**Figure 3**). However, urinary lead interactions were significantly associated with hypertension and diabetes (P < 0.05) (**Figure 4**). Increased urinary lead levels were positively associated with cancer in individuals with hypertension and diabetes (OR = 1.26 and OR = 1.41, respectively).

### The threshold between urine metals and cancer risk

**Figure 5:**
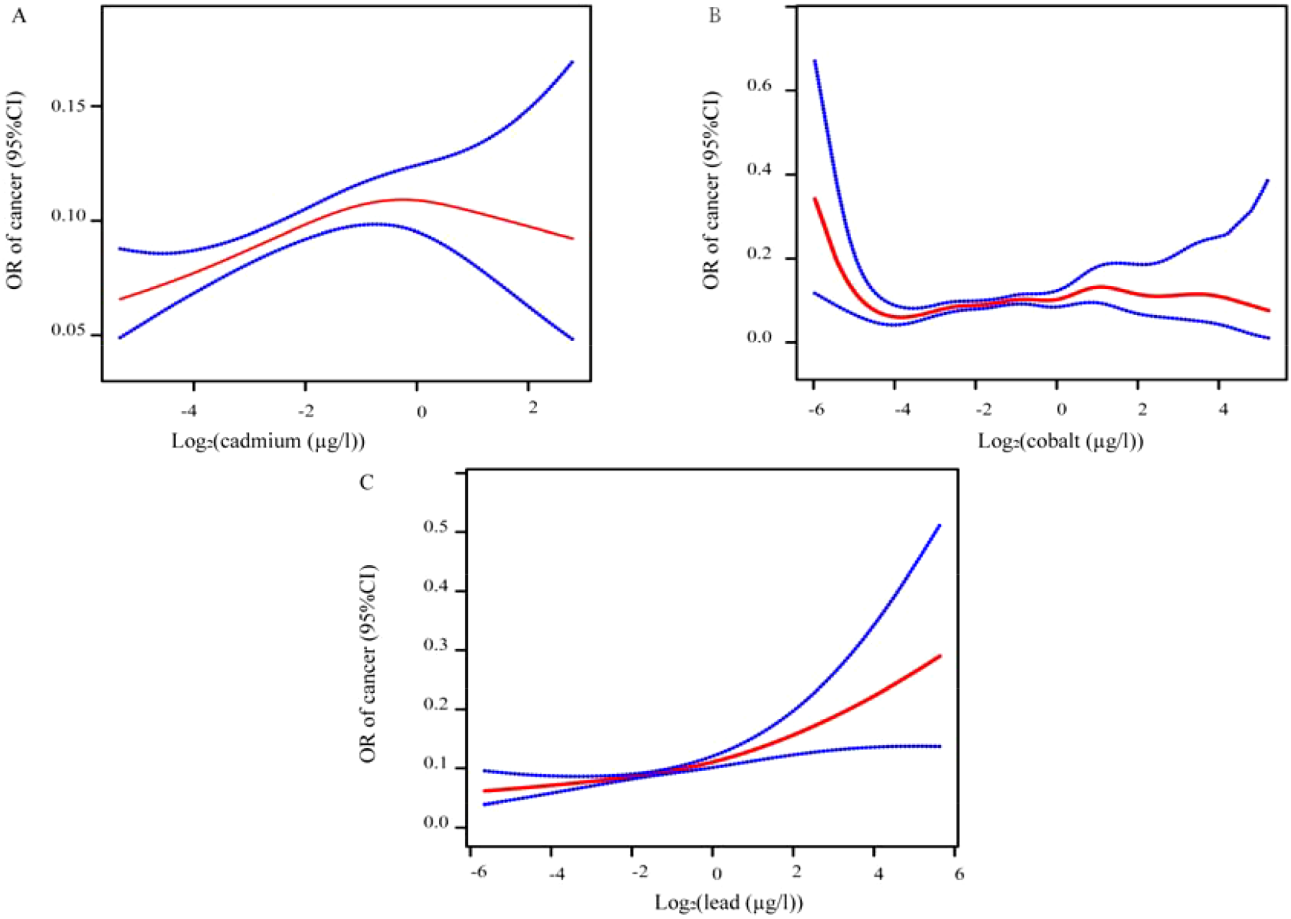
Log_2_-transformed urinary metal levels and cancer risk were plotted using restricted cubic spline (RCS). RCS regression was adjusted for gender, age, race, education level, BMI, hypertension, diabetes status, and PIR (Model 3). The red and blue lines represent the odds ratios and 95% confidence intervals. Specifically, **(A)** shows the relationship between log_2_-transformed cadmium and cancer risk; **(B)** shows the relationship between log_2_-transformed cobalt and cancer risk; **(C)** shows the relationship between log_2_-transformed lead and cancer risk.

We used smoothed plots to visualize the relation between urinary metals and the risk of cancer (**Figure 5**). A significant inverted U-shaped curve was observed between urinary cadmium concentration and tumor risk (P for non-linearity <0.001). The risk of cancer increased with urinary cadmium levels up to the turning point (log_2_-cadmium = −0.05, corresponding to cadmium = 0.97 µg/l). Here, “log_2_-cadmium” represents the logarithm of cadmium concentration to the base 2. However, after the turning point, the prevalence of cancer decreased as urinary cadmium levels increased. Likewise, there was a nonlinear association between log_2_-transformed urinary cobalt and the prevalence of cancer (P = 0.004). The risk of cancer remained relatively stable below 0.97 µg/l (log_2_-lead = −0.01) of urinary lead, after which it started to increase rapidly (P for non-linearity <0.0001).

Log_2_-transformed urinary metal levels and cancer risk were plotted using restricted cubic spline (RCS). RCS regression was adjusted for gender, age, race, education level, BMI, hypertension, diabetes status, and PIR (Model 3). The red and blue lines represent the odds ratios and 95% confidence intervals. Specifically, (A) shows the relationship between log_2_-transformed cadmium and cancer risk; (B) shows the relationship between log_2_-transformed cobalt and cancer risk; (C) shows the relationship between log_2_-transformed lead and cancer risk.

## Discussion

In this study, we observed that participants with higher urinary metals levels exhibited an increased risk of cancer. A sensitivity analysis confirmed the robustness of the results. The association was roughly similar in different populations with respect to gender, age, BMI, hypertension, and diabetes status. In addition, the association between urinary metals levels and the risk of cancer was nonlinear. Specifically, the cancer prevalence increased rapidly when urinary lead concentrations exceeded 0.97 µg/l. There was a need to consider urinary metal exposure in clinical settings when assessing people with cancer.

It is the first study to examine the relationship between multiple urinary heavy metal burdens and cancer risk. Several studies have found that metal exposure increases cancer risk (12–16). Zhang et al. reported that an increased risk of lung cancer was associated with higher blood Mo concentration (17). In a previous study, it was found that every doubling of Pb levels increased the odds of prostate cancer by 2.04-fold (5). In previous studies, increased blood cadmium concentration has been implicated in endometrial cancer research (18, 19). All of the above studies have shown that there is a significant association between metals exposure and an elevated risk of cancer among individuals. We found similar results in our study.

As a result of disrupting cellular homeostasis, inducing oxidative stress, and causing DNA damage, metals may contribute to the development of cancer (20, 21). Further, they may alter key signaling pathways that are involved in cell proliferation, epithelial-mesenchymal transitions (22, 23).A primary mechanism of lead (Pb) toxicity is oxidative stress. Pb triggers the production of reactive oxygen species (ROS) inside cells, overwhelming antioxidant defenses and causing oxidative damage to lipids, proteins, and DNA. This cascade results in cellular dysfunction, apoptosis, and ultimately, tissue damage. Cadmium (Cd) primarily elevates reactive oxygen species (ROS) production by inhibiting antioxidant enzymes like superoxide dismutase (SOD) and depleting glutathione (GSH). Consequently, the fundamental mechanism of Cd toxicity lies in the resulting imbalance between heightened oxidative stress and diminished detoxification capacity (6).

Overall, we found that urinary cadmium concentrations and cancer risk are positively correlated. Even though only 1–5% of ingested cadmium was absorbed by the body, its biological half-life was extremely long (24–26). Unbalanced detoxification and increased oxidative stress are the primary mechanisms of cadmium toxicity (27–29). Furthermore, cadmium falls into the category of endocrine disruptor chemicals, which mimic or block endogenous hormone activity (30). Cadmium can interfere with hormones, blocking their interactions with natural counterparts like estrogens and androgens, thus leading to improper hormone signaling (31, 32). Among common heavy metal pollutants, urine cadmium is unique in that it provides information about the total body burden of cadmium over a lifetime (33). The exact range of urinary cadmium that is considered safe has not yet been determined. A urinary cadmium level below 1.0 µg/g is associated with an increased risk of breast cancer, according to previous studies (34). According to our study, the risk of cancer increased with an increase in urinary cadmium concentration below 0.97 µg/l, but decreased with an increase above 0.97 µg/l. Our results may differ slightly from these results for the following reasons. Although urine cadmium levels closely parallel cadmium body burden until 50–60 years of age, recent exposures may also be reflected in urine cadmium among older general populations (35). Due to variable urinary dilution effects throughout the day, spot urine samples may limit the accuracy of exposure assessments (36).

In our study, when urine lead concentrations exceeded 0.97 µg/l, we observed a rapid increase in cancer prevalence. Similarly, a cohort of 20,700 Finnish lead - exposed workers demonstrated that high blood lead levels increased the overall cancer incidence and lung cancer incidence by 1.4 - and 1.8 - fold, respectively (37). The results of one study indicated a 6.60 - fold increase in cancer - specific mortality for individuals with high urinary lead levels when compared to those with low urinary lead levels (38). Our results are generally consistent with previous studies. Reactive oxygen species (ROS) are generated in cells by lead, which overwhelms the body's antioxidant defenses, resulting in oxidative damage to lipids, proteins, and DNA (2, 39–41). According to the nonlinear effects of urinary lead on cancer risk observed in our study, a urinary lead level lower than 0.97 µg/l may be considered relatively safe.

There are several strengths in our research. Firstly, NHANES data, obtained according to a standard procedure, served as the basis for our study. To ensure reliability, we adjusted for confounding factors, which were primarily selected based on previous studies assessing the relationship between cancer and other exposure variables. Additionally, in our study, urinary metals were used as the most non-invasive method for estimating metal concentrations in the body. Moreover, urinary metal concentration has been used as a biomarker for a wide range of physiological and pathological conditions, such as thyroid and renal dysfunctions, cardiac disease, etc (42–44).

However, the present study has several limitations that need to be addressed. The NHANES study used casual (spot) urine samples for the detection of urinary metal concentrations, without considering the effects of metal exposure over time. A second concern is that although confounders were adjusted, possible confounders, such as metals in excipients, may have remained unaccounted for. Due to its cross-sectional design, NHANES cannot establish a causal relationship between urinary metals and cancer. Therefore, it is necessary to confirm this association in future prospective studies.

## Conclusion

Increased urinary levels of metals such as cadmium, cobalt, and lead in the United States were associated with an elevated cancer risk, indicating that exposure to urinary metals has an adverse effect on cancer risk. We need to conduct further research to validate our results.

## Data Availability

The raw data supporting the conclusions of this article will be made available by the authors, without undue reservation.
